# Primary Malignant Melanoma of the Urethra: A Rare Finding

**DOI:** 10.7759/cureus.46462

**Published:** 2023-10-04

**Authors:** Nova B Mebane, Gerard Voorhees, Emile Salloum, Michael Bailey, Aaron Moon

**Affiliations:** 1 Internal Medicine, Corpus Christi Medical Center, Corpus Christi, USA; 2 Radiation Oncology, Corpus Christi Medical Center, Corpus Christi, USA; 3 Medical Oncology, Corpus Christi Medical Center, Corpus Christi, USA; 4 Pathology, Corpus Christi Medical Center, Corpus Christi, USA; 5 Radiology, Corpus Christi Medical Center, Corpus Christi, USA

**Keywords:** pd-l1 expression, primary metastatic melanoma of the urethra, sick sinus syndrome, nivolumab, ipilimumab

## Abstract

Primary urinary tract metastatic melanoma is an extremely rare cancer involving the urinary tract. In the majority of melanomas, skin melanocytes can become damaged by ultraviolet radiation and cause melanoma. However, in rare cases, melanocytes in mucosal tissue can develop into melanoma, which can be challenging to diagnose and treat. This case report describes managing and treating a patient with an extremely rare primary cancer finding. We describe the case of a patient who was diagnosed with primary metastatic melanoma of the urinary tract following an extensive and detailed diagnostic period of 2.5 months. After review by an interdisciplinary tumor board and a shared decision-making process with the patient, he agreed to immunotherapy with ipilimumab and nivolumab. The patient survived nearly five months after the initiation of treatment. The cause of death was challenging to determine due to it being unwitnessed; however, it is suspected to be cardiovascular-related, owing to a history of severe cardiovascular disease and an acute stress event prior to death.

## Introduction

Only 38 cases of primary bladder melanoma (PBM) have been reported since 2021 [1]. Although melanoma is commonly known for its cutaneous lesions, it can also present as mucosal lesions. The mucosal subtypes, in order of frequency, are found in the head and neck, anorectal region, vulvovaginal area, and urinary tract. PBM represents 0.2% of all melanomas and typically is diagnosed at a median age of 61 years [2]. There is currently no standardized management protocol specifically for PBM; therefore, previous cases have referred to treatment protocols for cutaneous disease, with limited success in reducing mortality [3]. A review of the literature demonstrates that the majority of patients had metastasis at initial diagnosis, a high likelihood of recurrence post-treatment, and poor prognosis once the tumor had invaded the muscle layer. Diagnosing PBM can be very difficult due to the necessity to rule out skin or visceral sources as a primary source. A pathological diagnosis of bladder melanoma involves a biopsy of the urinary tract system showing atypical melanocytes. Mucosal markers which could support the diagnosis include c-KIT, NF1, RAS, MART-1, MITF, HMB-45, S100, and SYR-box10 [1]. In addition to biopsy, patients suspected of PBM need to be evaluated by a dermatologist, and any suspicious lesion should be biopsied. A positron emission tomography (PET) scan is recommended to rule out other primary sources. There are two main theories on how mucosal melanoma can develop as a primary source in the urinary tract. The first theory is that melanoblasts migrate from the neural cusps into the mesenchyme during embryogenesis and localize in the urinary tract as ectopic tissue [1]. This can lead to transformation into malignancy later in life with possible chronic exposure to certain environmental factors that are unknown at this time. The second theory is that urothelial cells derived from stem cells differentiate into neoplastic melanocytes [1].

## Case presentation

**Figure 1:**
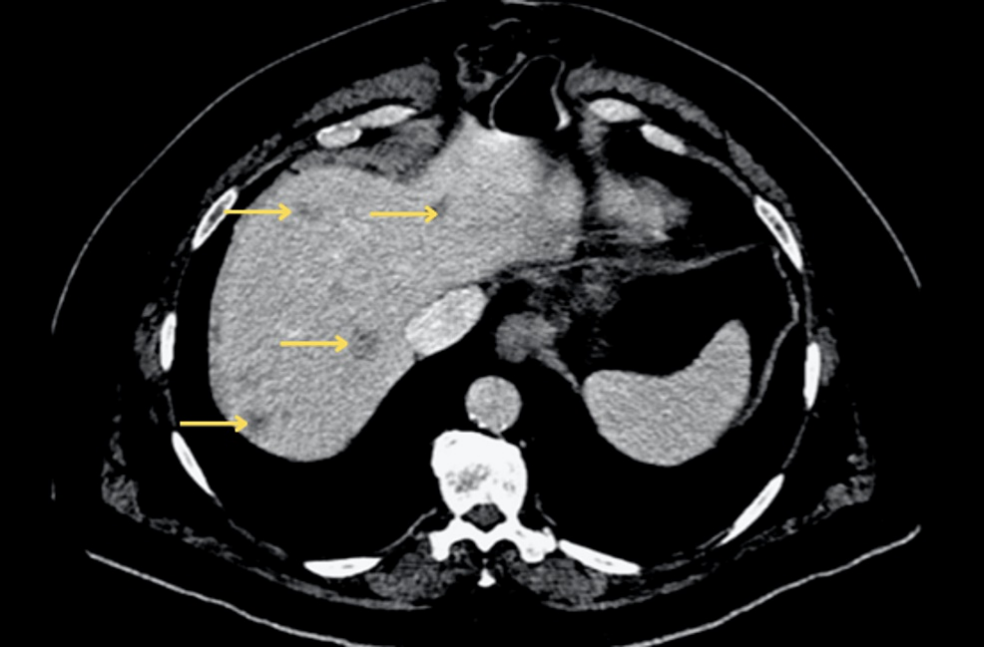
CT scan of the abdomen prior to immunotherapy. Arrows highlight a selection of numerous hypoenhancing liver lesions, indicative of metastasis.

**Figure 2:**
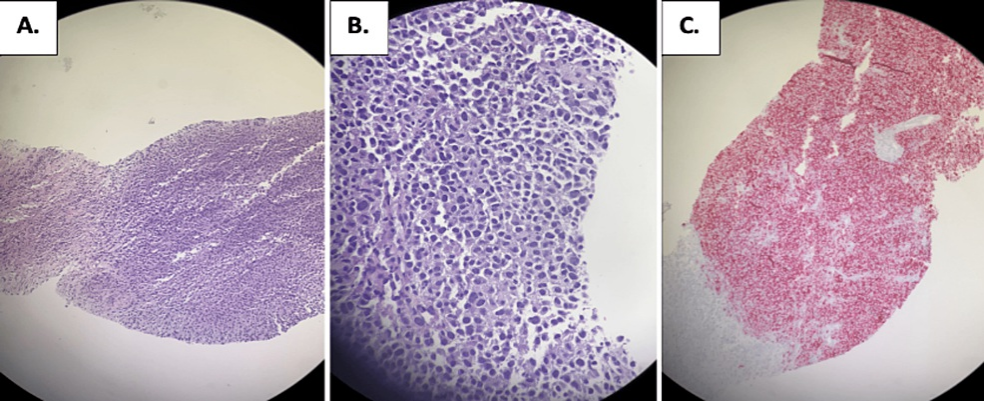
Histopathological slides from urethral biopsy of urothelial cells. A: The left portion of the slide displays normal urothelial cells, while the right reveals macronuclei and hypercellularity characteristic of tumor cells. B: A higher-power view of melanoma reveals loosely cohesive cells with large nuclei and an epithelioid-like character. C: SOX10 protein stain positive for melanoma, evidenced by the red-stained cytoplasm.

The patient was a 71-year-old male with a past medical history of longstanding type II diabetes mellitus, peripheral vascular disease, coronary artery disease status post coronary artery bypass graft surgery, hypertension, and dyslipidemia who presented to his primary care physician with gross hematuria. The patient was a former smoker and worked in the oil field industry. The patient’s complete blood count, creatinine, and electrolytes were all within normal range. The patient was referred to urology. An initial CT of the abdomen and pelvis (Figure 1) showed multiple hepatic lesions consistent with metastatic disease. The largest liver lesion measured 2.2 cm in segment VIII. Additionally, there was enlarged left inguinal lymphadenopathy measuring 4 x 2.2 cm. A cystoscopy was performed in which urothelial specimens and a bladder wash were obtained. The bladder wash pathology utilizing two cytospin slides (1 H&E, 1 Pap stain) showed high-grade urothelial carcinoma. A biopsy of the urethra was performed as well. The urothelial specimens (Figure 2) revealed malignant melanoma with cells positive for SOX10, HMB45, and Mart1 while negative for S100, GATA3, and pankeratin.

**Figure 3:**
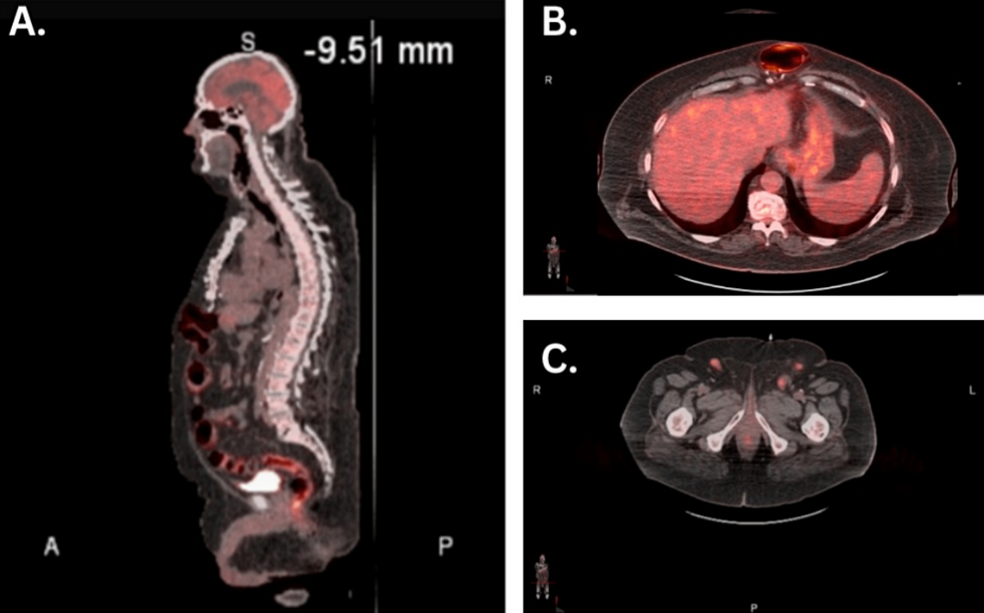
PET scan after immunotherapy treatment. A: Sagittal view. B: Transverse view showing the liver with normal radiotracer uptake. C: Transverse view with bilateral inguinal lymph nodes having high radiotracer uptake indicative of metastasis still present. PET: Positron emission tomography.

The lymph node biopsy showed metastatic melanoma positive for SOX10, HMB45, and Mart1 and was negative for GATA3, CK7, CK20, pankeratin, S100, CDX2, villin TTF-1, PSA, PAX8, arginase, CD56, synaptophysin, chromogranin, and CD45. A tumor board meeting was held, which included medical oncologists, a radiation oncologist, an interventional radiologist, a surgical oncologist, and a pathologist. Due to the metastatic melanoma result, the patient was dispositioned to begin ipilimumab and nivolumab every three weeks for a total of four cycles with a restaging PET scan after the completion of treatment (Figure 3). Surgical intervention or radiotherapy was deemed unsuitable due to the tumor's location and the presence of metastasis. Additionally, a dermatology evaluation revealed no suspicious cutaneous lesions indicative of melanoma, thereby ruling out the possibility of primary skin cancer with metastasis to the urethra.

Additional imaging, which included a CT of the brain and thorax with contrast, revealed no evidence of metastasis or lesions in either area. A genetic analysis was conducted to explore novel therapeutic options, with the biomarker analysis presented in Table 1. Notably, BRAF was not detected; PD-L1 (SP142) was positive at 1%; TrkA/B/C was positive at 2%; and ARID2 was labeled as a 'variant of uncertain significance.' The microsatellite instability biomarker was deemed 'stable,' tumor mutational burden was 'low, five,' and genomic loss of heterozygosity was high (22%). Although the patient was informed about available clinical trials, he opted for treatment with nivolumab and ipilimumab.

**Table 1:** Cancer-type relevant biomarker results.

Biomarker	Method	Analyte	Result
PD-L1 (SP142)	IHC	Protein	Positive, 1+, 1%
PTEN	Seq	DNA-Tumor	Pathogenic variant Exon 8, p.W274
	CNA-Seq	DNA-Tumor	Deletion Not Detected
TrkA/B/C	IHC	Protein	Positive, 1+, 2%
MSI	Seq	DNA-Tumor	Stable
Mismatch Repair Status	IHC	Protein	Proficient
NTRK1	Seq	RNA-Tumor	QNS
NTRK2	Seq	RNA-Tumor	QNS
NTRK3	Seq	RNA-Tumor	QNS
Tumor Mutational Burden	Seq	DNA-Tumor	Low, 5 mut/Mb
ARID2	Seq	DNA-Tumor	Variant of Uncertain Significance Exon 4, p.I124V
BRAF	Seq	RNA-Tumor	QNS
	CNA-Seq	DNA-Tumor	Amplification Not Detected
CDKN2A	CNA-Seq	DNA-Tumor	Deletion Not Detected
	Seq	DNA-Tumor	Mutation Not Detected
KIT	CNA-Seq	DNA-Tumor	Amplification Not Detected
	Seq	DNA-Tumor	Mutation Not Detected
MAP2K1 (MEK1)	Seq	DNA-Tumor	Mutation Not Detected
MAP2K2 (MEK2)	Seq	DNA-Tumor	Mutation Not Detected
MTAP	CNA-Seq	DNA-Tumor	Deletion Not Detected
NF1	CNA-Seq	DNA-Tumor	Deletion Not Detected
	Seq	DNA-Tumor	Mutation Not Detected
NRAS	Seq	DNA-Tumor	Mutation Not Detected
RAC1	Seq	DNA-Tumor	Indeterminant
KMT2D	Seq	DNA-Tumor	Pathogenic Variant Exon 34I p.G2892fs
FANCM	CNA-Seq	DNA-Tumor	Deleted

He responded well to dual immunotherapy with ipilimumab and nivolumab regimen. He denied fevers, chills, and diarrhea with no reported significant side effects. The restaging PET scan post immunotherapy showed no change in the left inguinal lymph node; however, there was significant regression of liver metastasis. Liver lesions were not seen on the non-contrast CT scan post-treatment. The left inguinal lymph node was reduced in size and soft in texture on the physical exam. He continued single-agent nivolumab. The patient was briefly hospitalized for fatigue symptoms with brown urine for one week. Cardiac catheterization was performed one month before his death, indicating hemodynamically stable severe cardiovascular disease, which was present before the start of immunotherapy. The cardiologist recommended continued immunotherapy for malignant melanoma and noted the prognosis as "guarded." The patient was pronounced deceased 25 days after his last cardiac catheterization.

## Discussion

Our patient was diagnosed with stage IV urethral melanoma metastatic to the liver and left inguinal lymph node. His cancer was positive for PDL-1 expression without BRAF mutation and negative NTRK1, 2, and 3. This is considered an exceptionally rare finding for melanoma to be primarily found in urothelial mucosal tissue. It took approximately 10 weeks to properly diagnose this patient due to the necessity to exclude other possible primary cancer sites and obtain imaging/biopsy results. The patient survived almost five months after the initiation of treatment. There was notable regression of liver lesions on the dual immunotherapy regimen of ipilimumab and nivolumab. The cause of death was presumed to be cardiovascular-related; however, it was unwitnessed. Known cardiovascular adverse reactions for ipilimumab include hypertension (9%) and myocarditis (less than 1%) [4], and for nivolumab, there are reported adverse events of acute coronary syndrome (ACS), vasculitis, myocarditis, and pericarditis [5]. Nivolumab is an immune checkpoint inhibitor that can cause instability of atherosclerotic lesions, leading to ACS [6]. Of note, there were no findings prior to his death of myocarditis or pericarditis on electrocardiogram or echocardiogram. Additionally, a cardiac catheterization performed prior to death showed patent graft vessels. The patient had a complex history of coronary artery disease with coronary artery bypass surgery, which was stable at the time of cardiac catheterization. Unfortunately, he suffered the loss of an immediate family member approximately two weeks before his death.

When managing PBM, there needs to be an individualized approach with three main factors of consideration. First, this specific cancer metastasizes rapidly, with an overall survival rate of three years [3]. Time from symptomatic onset to treatment is crucial [7]. This patient was diagnosed approximately 10 weeks from the onset of hematuria and began immunotherapy approximately three months after the onset of hematuria. Second, the results of the staging exams are essential for proper treatment selection. Surgery should be considered if the cancer is localized. The third factor to consider is whether the patient is a candidate for immunotherapy treatment, including patients with no autoimmune disease, no use of immunosuppressive therapy/corticosteroids, and no medical comorbidities complicating management. Currently, the first-line therapy for BRAF-mutated metastatic melanoma is immunotherapy due to superior long-term outcomes as opposed to BRAF and MEK inhibitors [7]. Prior to targeted therapy, immunotherapy is recommended, especially in treatment-naive patients [7]. Even with localized early-stage PBM, there has been minimal success regarding long-term survival. A case study by Mercimek MN and Ozden E had localized bladder cancer invading the detrusor muscle with no spread to the pelvic lymph nodes. A laparoscopic partial cystectomy with bilateral pelvic lymph node dissection was the only treatment performed because the patient declined any further treatment. Unfortunately, the cancer recurred six weeks post-op, with death three months later due to cranial metastasis. It is important to note that all the treatment regimens discussed here are for the management of metastatic melanoma and are mainly from studies and guidelines of cutaneous disease. However, one study by Huang et al. revealed five possible predisposing genes (ARID1B, catalase, EIF4G3, ANK3A, and collagen type I) and two driver genes (PTEN and AHANK) which may be responsible for primary malignant transformation in the urethra. Specific targeting of these genes may prove to be efficacious in survival outcomes [8].

## Conclusions

This case study demonstrates a rare finding of primary urinary tract melanoma. Using detailed imaging, biopsy, and interdepartmental collaboration, the patient was able to tolerate 12 weeks of immunotherapy with significant metastasis regression. Our goal was to explain our management and outcomes of this rare disease in the hope that it may benefit healthcare providers with patients with a similar clinical presentation.
